# Unraveling unique features of plasma cell clones in POEMS syndrome with single-cell analysis

**DOI:** 10.1172/jci.insight.151482

**Published:** 2022-10-24

**Authors:** Yusuke Isshiki, Motohiko Oshima, Naoya Mimura, Kensuke Kayamori, Yurie Miyamoto-Nagai, Masahide Seki, Yaeko Nakajima-Takagi, Takashi Kanamori, Eisuke Iwamoto, Tomoya Muto, Shokichi Tsukamoto, Yusuke Takeda, Chikako Ohwada, Sonoko Misawa, Jun-ichiro Ikeda, Masashi Sanada, Satoshi Kuwabara, Yutaka Suzuki, Emiko Sakaida, Chiaki Nakaseko, Atsushi Iwama

**Affiliations:** 1 Department of Hematology, Chiba University Hospital, Chiba, Japan.; 2 Division of Stem Cell and Molecular Medicine, Center for Stem Cell Biology and Regenerative Medicine, The Institute of Medical Science, The University of Tokyo, Tokyo, Japan.; 3 Department of Transfusion Medicine and Cell Therapy, Chiba University Hospital, Chiba, Japan.; 4 Laboratory of Systems Genomics, Department of Computational Biology and Medical Sciences, Graduate School of Frontier Sciences, The University of Tokyo, Tokyo, Japan.; 5 Department of Advanced Diagnosis, Clinical Research Center, National Hospital Organization Nagoya Medical Center, Nagoya, Japan.; 6 Department of Hematology, International University of Health and Welfare, Chiba, Japan.; 7 Department of Neurology, Chiba University Graduate School of Medicine, Chiba, Japan.; 8 Department of Pathology, Chiba University Hospital, Chiba, Japan.; 9 Laboratoty of Cellular and Molecular Chemistry, Graduate School of Pharmaceutical Sciences, The University of Tokyo, Tokyo, Japan.

**Keywords:** Hematology, Bone marrow, Clonal selection, Lymphomas

## Abstract

POEMS syndrome is a rare monoclonal plasma cell disorder, with unique symptoms distinct from those of other plasma cell neoplasms, including high serum VEGF levels. Because the prospective isolation of POEMS clones has not yet been successful, their real nature remains unclear. Herein, we performed single-cell RNA-Seq of BM plasma cells from patients with POEMS syndrome and identified POEMS clones that had Ig λ light chain (*IGL*) sequences (*IGLV1-36*, *-40*, *-44*, and -*47*) with amino acid changes specific to POEMS syndrome. The proportions of POEMS clones in plasma cells were markedly smaller than in patients with multiple myeloma (MM) and patients with monoclonal gammopathy of undetermined significance (MGUS). Single-cell transcriptomes revealed that POEMS clones were CD19^+^, CD138^+^, and MHC class II^lo^, which allowed for their prospective isolation. POEMS clones expressed significantly lower levels of *c-MYC* and *CCND1* than MM clones, accounting for their small size. *VEGF* mRNA was not upregulated in POEMS clones, directly indicating that VEGF is not produced by POEMS clones. These results reveal unique features of POEMS clones and enhance our understanding of the pathogenesis of POEMS syndrome.

## Graphical abstract

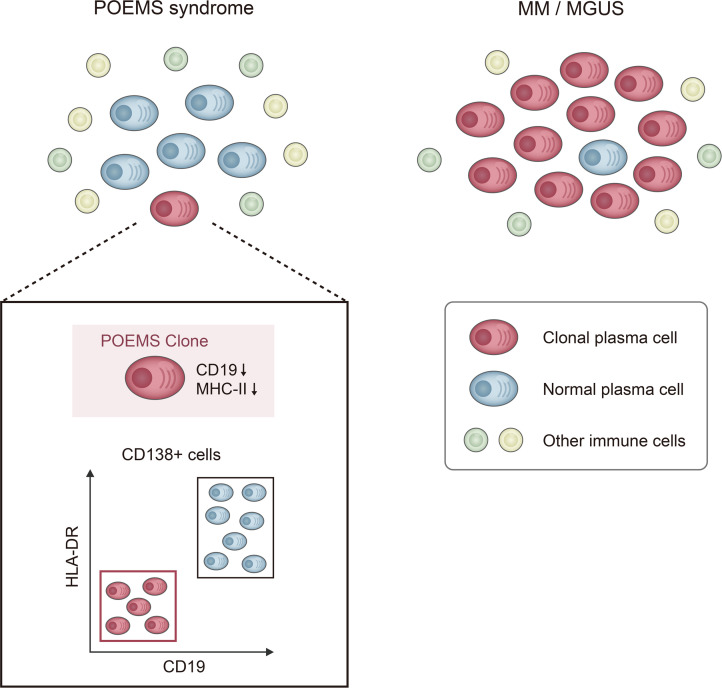

## Introduction

Polyneuropathy, organomegaly, endocrinopathy, monoclonal gammopathy or M protein, and skin changes (POEMS) syndrome, also known as Takatsuki disease or Crow-Fukase syndrome, is a rare disease initiated by monoclonal plasma cells (1–3). Serum levels of VEGF are elevated in POEMS syndrome; thus, they are one of the main diagnostic criteria (4, 5) and are used as a biomarker of the disease state (6, 7).

In contrast to the clinical advances in diagnostic and therapeutic strategies, limited information is currently available on the pathogenesis of POEMS syndrome. Plasma cells in POEMS syndrome account for approximately 2%–5% of total BM nucleated cells (8, 9), which is markedly lower than that in multiple myeloma (MM) (10). M protein is essentially restricted to the Ig λ light chain (IGL) in POEMS syndrome (9, 11), whereas MM and monoclonal gammopathy of undetermined significance (MGUS) are not. In our previous studies and subsequent studies by other groups, variable regions from the clonal *IGL* (*IGLV*) were found to be restricted to *IGLV1-44* or *IGLV1-40* germline sequences in POEMS syndrome (12–15), suggesting that clonal plasma cells lack diversity in the Ig repertoire. Half of the cases examined did not show the significant enrichment of POEMS-specific *IGLV* sequences in our genomic sequencing of BM mononuclear cells, indicating a small clone size in these cases (14). Our recent genome sequencing of BM plasma cells in POEMS syndrome identified 7 recurrently mutated genes: *KLHL6*, *LTB*, *EHD1*, *EML4*, *HEPHL1*, *HIPK1*, and *PCDH10* (16). Most of them were also identified in another independent study (17). Importantly, none of the driver gene mutations frequently found in MM were identified in either of these studies, suggesting that the mutational characteristics of POEMS syndrome are different from those of other plasma cell neoplasms. Moreover, transcriptome profiles of BM plasma cells in POEMS syndrome also showed a unique signature that was distinct from those in MM and MGUS (16). However, monoclonal plasma cells from patients with POEMS syndrome (referred to here in as POEMS clones) have not yet been identified because of their small size, and thus, their characteristics remain obscure.

To clearly identify POEMS clones and clarify their transcriptional signatures, we herein performed single-cell RNA-Seq (scRNA-Seq) using purified CD138^+^ plasma cells from BM and profiled the Ig repertoire of each single plasma cell. This is the first study to our knowledge that successfully identified POEMS clones and characterized their unique transcriptional signatures using scRNA-Seq analyses.

## Results

### Detection of POEMS clones by scRNA-Seq.

**Figure 1:**
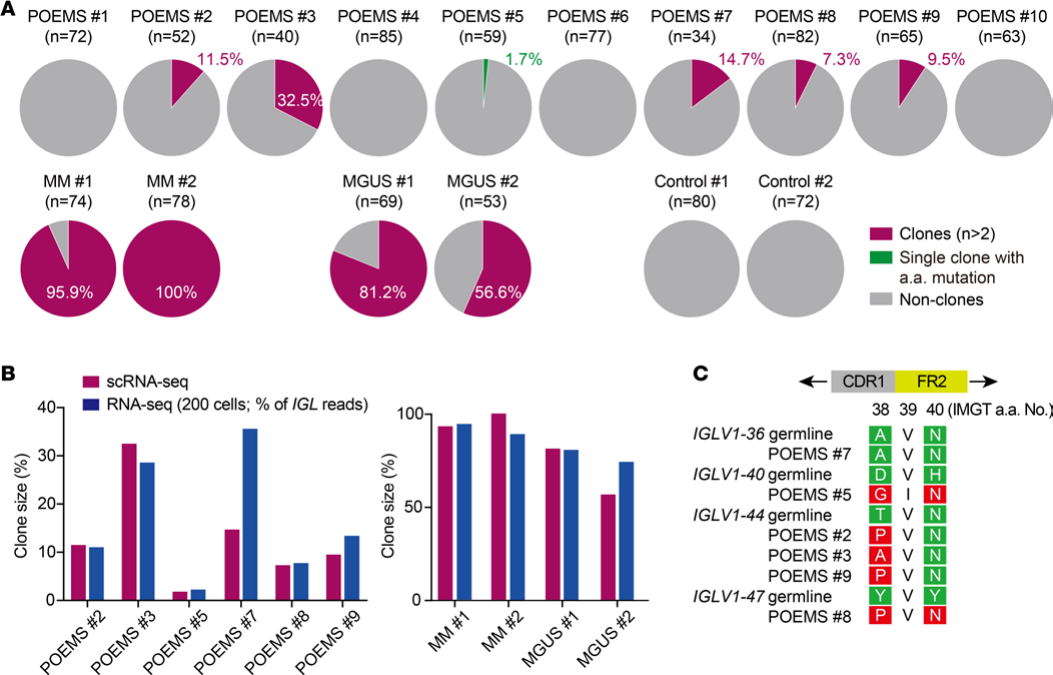
Identification of clonal plasma cells in POEMS syndrome. (**A**) *IGL* repertoire analysis of plasma cells in POEMS syndrome. The proportions of clonal plasma cells identified by scRNA-Seq are depicted in pie charts. Plasma cells with the same V-(D)-J and CDR3 sequences of the *IGL* and *IGH* genes were defined as clonal plasma cells. Numbers in parentheses are the single plasma cell numbers captured by Fluidigm C1. Magenta, clones; gray, nonclones; green, a single plasma cell identified as a clone by its specific amino acid mutations in **C**. (**B**) Comparison of the proportion of clones estimated by scRNA-Seq and RNA-Seq of 200 plasma cells. Maroon bars show the proportions of clonal plasma cells identified by scRNA-Seq. Blue bars represent the frequencies of clonal *IGL* sequence reads in all *IGL* reads identified by RNA-Seq. (**C**) POEMS-specific amino acid mutations in the monoclonal IGL VJ domains. Mutated amino acids at positions 38 and 40 (CDR1 and FR2 regions, respectively) are highlighted in red. Germline sequences are highlighted in green. Amino acids are aligned according to international ImMunoGeneTics information system numbering (http://www.imgt.org).

BM CD138^+^ plasma cells from 10 newly diagnosed or relapsed patients with POEMS syndrome, including a variant with undetectable monoclonal gammopathy (18); newly diagnosed patients with MM and MGUS (2 patients each); and 2 patients with early-stage lymphoma without BM invasion as controls were subjected to scRNA-Seq in this study (Supplemental Tables 1 and 2; supplemental material available online with this article; https://doi.org/10.1172/jci.insight.151482DS1). We used the Fluidigm C1 platform, which covers full-length transcriptomes, to obtain the full sequences of Ig heavy and light chains (*IGH* and *IGL/K*). Because plasma cells express high levels of Ig gene mRNA, a scRNA-Seq analysis readily identified their sequences. Plasma cells with the same V-(D)-J and CDR3 sequences in the *IGL* and *IGH* genes were defined as clonal plasma cells. The majority of plasma cells from patients with MM and MGUS were clonal, whereas clonal plasma cells were not identified in the controls (Figure 1A and Supplemental Table 3). In patients with POEMS syndrome, all M protein^+^ patients had λ-type M protein, and BM plasma cells accounted for approximately 5% of BM mononuclear cells, which was lower than that in MM patients. Two patients (patients no. 7 and no. 9 with POEMS) were in relapse after autologous peripheral blood stem cell transplantation (Supplemental Table 1). POEMS clones were successfully identified in 5 of 10 patients with POEMS syndrome (patients no. 2, 3, and 7–9 with POEMS) (Figure 1A). As expected, the size of POEMS clones in all plasma cells was markedly smaller (1.7%–32.5%) than that of those from patients with MM (96% and 100%) and MGUS (57% and 81%) (Figure 1A and Supplemental Table 3). Clonal *IGLV* genes were strictly derived from *IGLV1-36* (patient no. 7 with POEMS) and *IGLV1-44* (patients no. 2, 3, and 9 with POEMS), which was consistent with previous findings (16). The *IGLV1-47* gene was also detected in a patient (patient no. 8 with POEMS) (Supplemental Table 3); this had previously been identified in our RNA-Seq analysis (16). All POEMS clones used the IGLJ3*02 gene in rearrangements (Supplemental Table 3), as previously reported (16). We then profiled the *IGL* repertoire by RNA-Seq of 200 plasma cells to validate the size of POEMS clones. The frequencies of clonal *IGL* sequences in 200 plasma cells was closely correlated with those of POEMS clones identified in scRNA-Seq (Figure 1B).

Amino acid changes in the CDR1 and FR2 regions of *IGLV1-40* (D to G and H to N) and *IGLV1-44* (T to P/A) are highly conserved in POEMS syndrome (15). We confirmed these changes in patients with *IGLV1-40* and *IGLV1-44* (Figure 1C and Supplemental Table 4). We also identified amino acid changes at positions 38 and 40 of *IGLV1-47* (Y to P and Y to N, respectively) (Figure 1C and Supplemental Table 4); this further supported the specificity of amino acid changes at positions 38 and 40 of *IGLV* in POEMS syndrome. scRNA-Seq also identified a single plasma cell with POEMS syndrome–specific *IGLV1-40* (1.7%) in patient no. 5 with POEMS, which had the typical amino acid changes at positions 38 and 40 (D to G and H to N, respectively) conserved among patients with POEMS syndrome (Figure 1, A and C, and Supplemental Table 4) (15). RNA-Seq of 200 plasma cells revealed that the proportion of *IGLV1-40* in patient no. 5 with POEMS was 2.2% (Figure 1B). These results indicated that patient no. 5 with POEMS had a clone with a very small size of approximately 2%. Even in other patients without detectable POEMS clones (patients no. 1, 4, 6, and 10 with POEMS), the RNA-Seq of 200 plasma cells detected *IGL* sequences compatible with POEMS syndrome (*IGLV1-40/44/47*) at low frequencies (1%–5%) (Supplemental Figure 1); however, it currently remains unclear whether they represent POEMS clones.

### Identification of unique gene expression patterns in POEMS clones.

**Figure 2:**
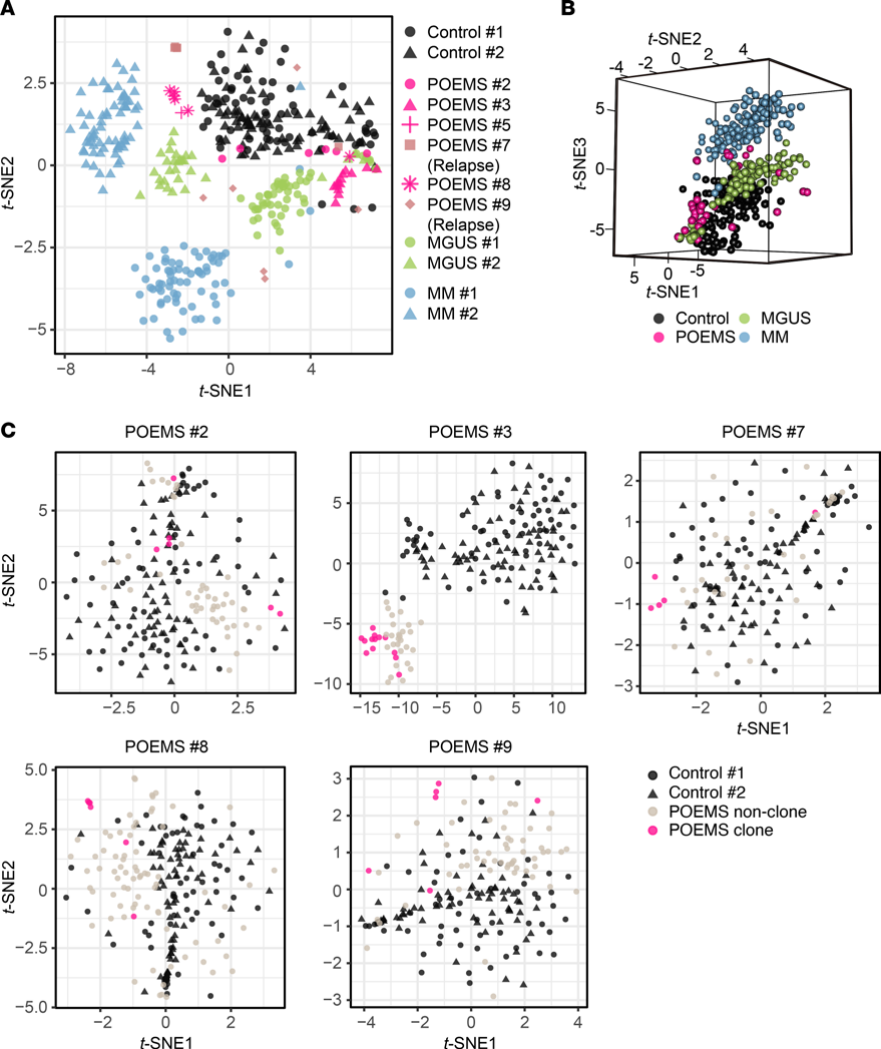
Transcriptional characteristics of POEMS clones. (**A** and **B**) *t*-SNE of the scRNA-Seq data of plasma cells from controls (black), POEMS clones (pink), MGUS clones (green), and MM clones (blue) depicted in (**A**) 2D and (**B**) 3D. (**C**) *t*-SNE of the scRNA-Seq data of plasma cells from controls (black), POEMS nonclones (light brown), and POEMS clones (pink).

To elucidate transcriptional features specific to POEMS clones, we first examined the presence of chromosomal translocations involving the *IGH* gene using scRNA-Seq data and identified t(11;14), which involves the *CCND1* gene in MGUS clone 1 (Supplemental Table 5). We then performed detailed profiling of transcriptomes in POEMS clones identified by the scRNA-Seq analysis. *t*-Distributed stochastic neighbor embedding (*t*-SNE) revealed that POEMS clones had unique expression profiles, which were different from those of MM clones, but were in between those of controls and MGUS clones (Figure 2, A and B). We next evaluated the relationship between POEMS clones and nonclones in each patient using the scRNA-Seq data. Similar to that in Figure 2, A and B, POEMS clones in each patient, except for patient no. 2 with POEMS, showed distinct expression signatures compared with nonclones and controls (Figure 2C). Although the gene expression profiles of nonclones were close to those of controls, nonclones from patient no. 3 with POEMS showed unique expression, and nonclones from patients no. 2, 8, and 9 with POEMS also exhibited mild differences in expression compared with controls, suggesting that POEMS syndrome–related symptoms, such as hypercytokinemia, endocrinopathy, or systemic inflammation, affect the phenotypes of nonclones.

**Figure 3:**
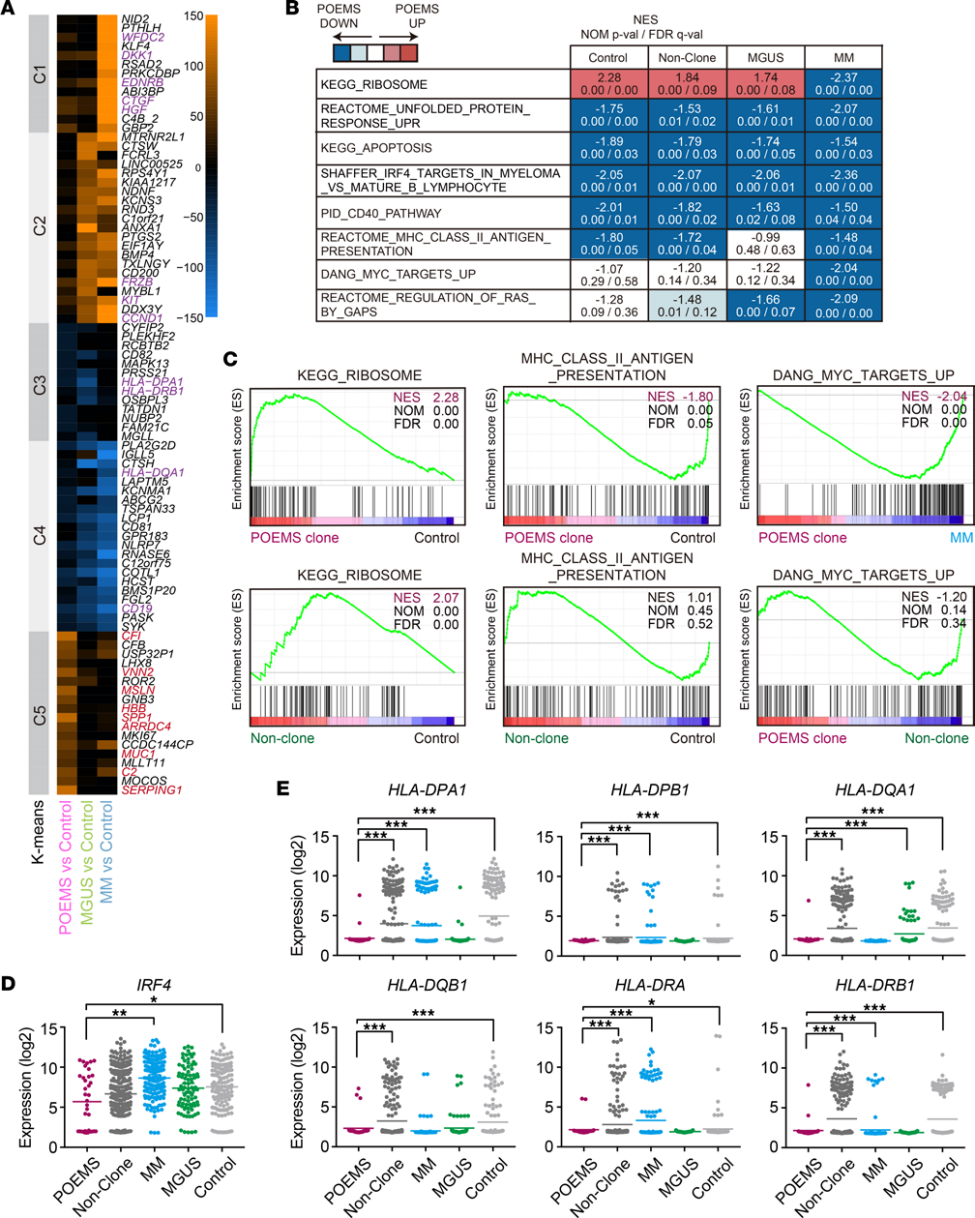
Transcriptional characteristics of POEMS clones. (**A**) K-means clustering of scRNA-Seq data. The top 30 genes upregulated and downregulated in POEMS, MGUS, and MM clones relative to those in control plasma cells (*P* < 0.01) were pooled (a total of 86 genes) and analyzed by K-means clustering. (**B**) Summary of gene set enrichment analysis (GSEA) data that showed positive (shown in red) or negative (shown in blue) enrichment in POEMS clones from control plasma cells, POEMS nonclonal plasma cells, MGUS clones, and MM clones. Normalized enrichment scores (NES), nominal *P* values (NOM-p), and false discovery rates (FDR-q) are indicated. (**C**) GSEA plots of representative gene sets. (**D**) *IRF4* expression levels in each plasma cell group in normalized read counts (log_2_). (**E**) MHC class II expression levels in normalized read counts (log_2_). Adjusted *P* values: *P < 0.05; **P < 0.01; ***P < 0.001 by DESeq2.

We then defined differentially expressed genes (DEGs) among clones from patients with POEMS, MGUS, or MM and control plasma cells using the cutoff value of *P* < 0.01 (Supplemental Tables 6 and 7). We selected the top 30 genes up- and downregulated in POEMS, MGUS, and MM clones, respectively, and pooled them (a total of 86 genes). We then analyzed their expression profiles by K-means clustering, which revealed a differential expression profile for POEMS clones compared with those for MGUS and MM clones (Figure 3A and Supplemental Figure 2). POEMS clones from relapse cases (no. 7 and no. 9) also showed similar expression profiles compared with newly diagnosed cases (Supplemental Figure 2). A gene set enrichment analysis (GSEA) revealed the positive enrichment of the ribosomal protein gene set in POEMS clones compared with those in controls and POEMS nonclones (Figure 3, B and C, and Supplemental Table 8). GSEA also revealed the negative enrichment of gene sets associated with unfolded protein response (UPR), apoptosis, IRF4 target genes, CD40 signaling, MHC class II (MHC-II), and MYC targets in POEMS clones compared with those in controls, POEMS nonclones, MGUS clones, and/or MM clones (Figure 3, B and C). The expression of *IRF4*, a master regulator of plasma cell differentiation (19), was significantly downregulated in POEMS clones (Figure 3D). Although the transcriptional activator of MHC-II genes, *CIITA* was expressed normally in POEMS clones (Supplemental Figure 3A), MHC-II, but not MHC-I, genes were generally downregulated in POEMS clones (Figure 3E and Supplemental Figure 3, A and B). As expected from the data in Figure 2C, GSEA revealed positive enrichment of several gene sets, such as ribosomal protein and inflammatory responses, in POEMS nonclones compared with control plasma cells, suggesting that nonclonal plasma cells are exposed to systemic inflammation (Figure 3C and Supplemental Table 8). In contrast, the levels of MHC-II gene expression were comparable between POEMS nonclones and control plasma cells, indicating that the downregulation of MHC-II genes is specific to POEMS clones.

### DEGs in POEMS clones.

**Figure 4:**
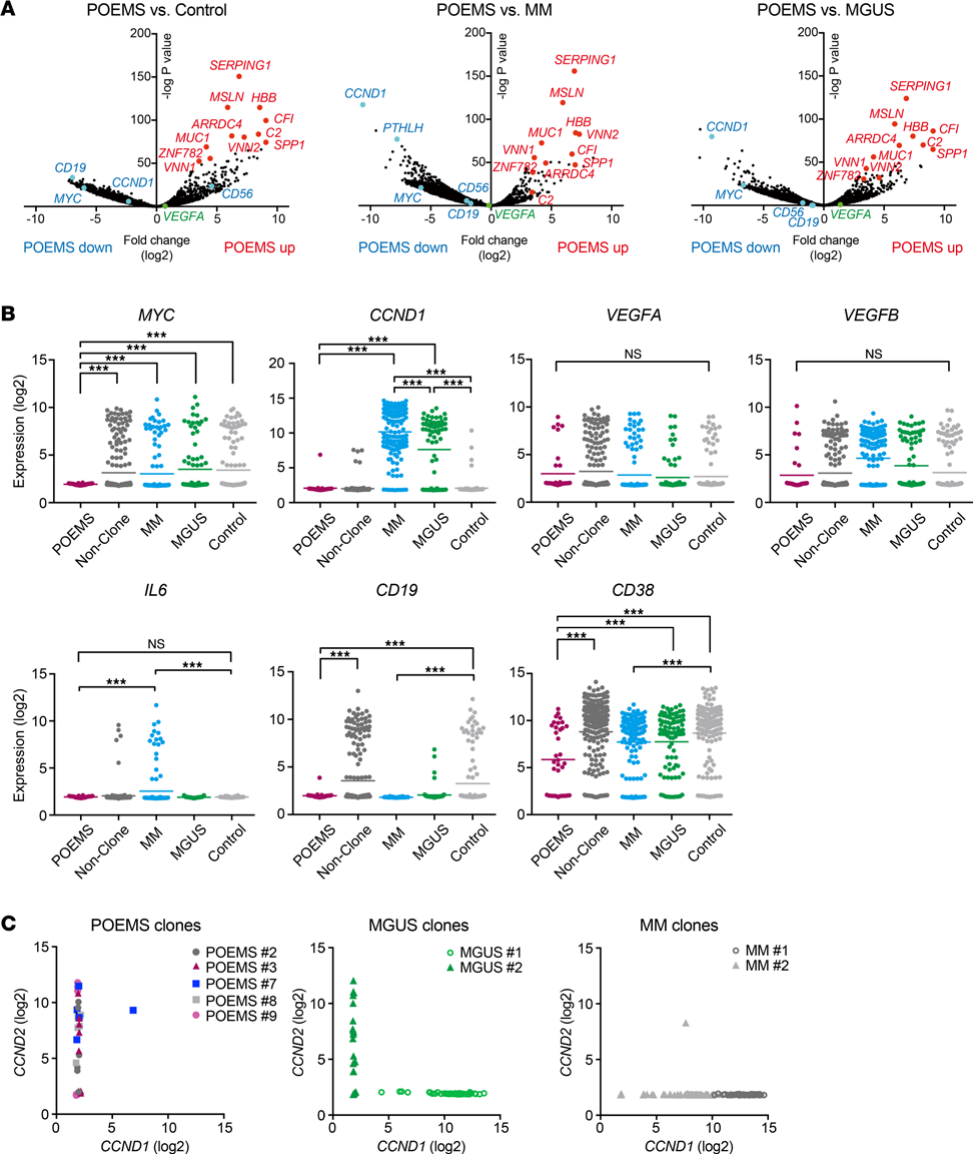
Profiling of the POEMS clone–specific gene signature. (**A**) Volcano plots of differentially expressed genes normalized by DESeq2. Genes upregulated and downregulated in POEMS clones are shown in red and blue, respectively. (**B**) Expression of genes associated with the pathogenesis of POEMS syndrome or MM. The signal levels of the genes in normalized read counts (log_2_) are shown. Mean values are indicated by horizontal bars. Adjusted *P* values: ***P < 0.001 by DESeq2. (**C**) Comparison of *CCND1* and *CCND2* expression for each clonal plasma cell. The signal levels of the genes in normalized read counts (log_2_) are shown.

We then focused on genes with dysregulated expression specific to POEMS clones. Functional profiling of DEGs in POEMS clones compared with control plasma cells revealed positive enrichment of gene ontology (GO) terms, such as viral transcription and protein localization and transport (Supplemental Table 9). A similar trend was also observed in POEMS clones compared with POEMS nonclones. In contrast, MHC-II–related GO terms were negatively enriched in POEMS clones (Supplemental Table 10). Volcano plots of scRNA-Seq data normalized by DESeq2 revealed that expression levels of *MYC* and *CCND1*, which are closely associated with the pathogenesis of MM (19, 20), were significantly lower in POEMS clones than in MM clones (Figure 4, A and B). Comparison of *CCND1* and *CCND2* in expression for each clonal plasma cell revealed that MM clones and MGUS clone no. 1 with t (11;14) expressed high levels of *CCDN1*, while POEMS clones expressed low levels of *CCDN1* (Figure 4C and Supplemental Figure 4). Detection of chromosomal translocations by screening scRNA-Seq data identified no other major translocations in POEMS clones (Supplemental Table 5). Correspondingly, POEMS clones did not show significant upregulation of *CCND3*, *MAF*, *MAFA*, *MAFB*, *NSD2*, or *FGFR3*, which are involved in MM-related translocations (data not shown).

The expression of several growth signaling genes, including *CD117* (also known as *KIT*) and *HGF*, was weaker in POEMS clones than in MM clones (Supplemental Figure 5A). Gene expression profiles of cell surface marker genes revealed that POEMS clones were CD19^–^ and expressed lower levels of CD38 than control plasma cells, similar to MM clones (Figure 4, A and B) (21, 22). *IL6*, which encodes one of the autocrine cytokines for MM clones (23), was not upregulated in POEMS clones (Figure 4B). Of note, *VEGF* mRNA was not upregulated in POEMS clones (Figure 4, A and B). We also identified upregulated genes which were specific to POEMS clones, such as *SPP1*, *SERPING1*, *CFI*, *C2*, *MSLN*, and *MUC1* (Figure 3A, Figure 4A, and Supplemental Figure 5B). Among the DEGs unique to POEMS clones described above, expression of *MYC*, *CCND1*, *CD38*, *CD19*, *VEGF*, and *IL6* showed similar trends in all POEMS clones, whereas expression of *SPP1*, *SERPING1*, *CFI*, *C2*, and *MUC1* was variable among POEMS clones (Supplemental Figure 6).

**Figure 5:**
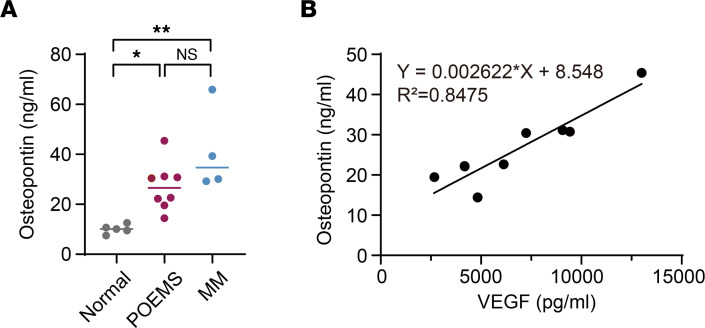
Serum levels of OPN in POEMS syndrome. (**A**) Serum levels of OPN in newly diagnosed patients with POEMS syndrome (*n* = 8) and MM (*n* = 4), and healthy volunteers (*n* = 5). Mean values are indicated by horizontal bars. *P < 0.05, **P < 0.01 by 1-way ANOVA. (**B**) Scatter plots and a simple linear regression line, showing the relationship between serum levels of OPN and VEGF in patients with POEMS syndrome (*n* = 8).

Of interest, while *SPP1* expression was upregulated only in a part of POEMS clones (Supplemental Figure 6), serum levels of osteopontin (OPN), encoded by *SPP1*, were significantly elevated in all patients with POEMS syndrome and well correlated with serum levels of VEGF (Figure 5).

### Discovery of POEMS clone–specific surface markers.

**Figure 6:**
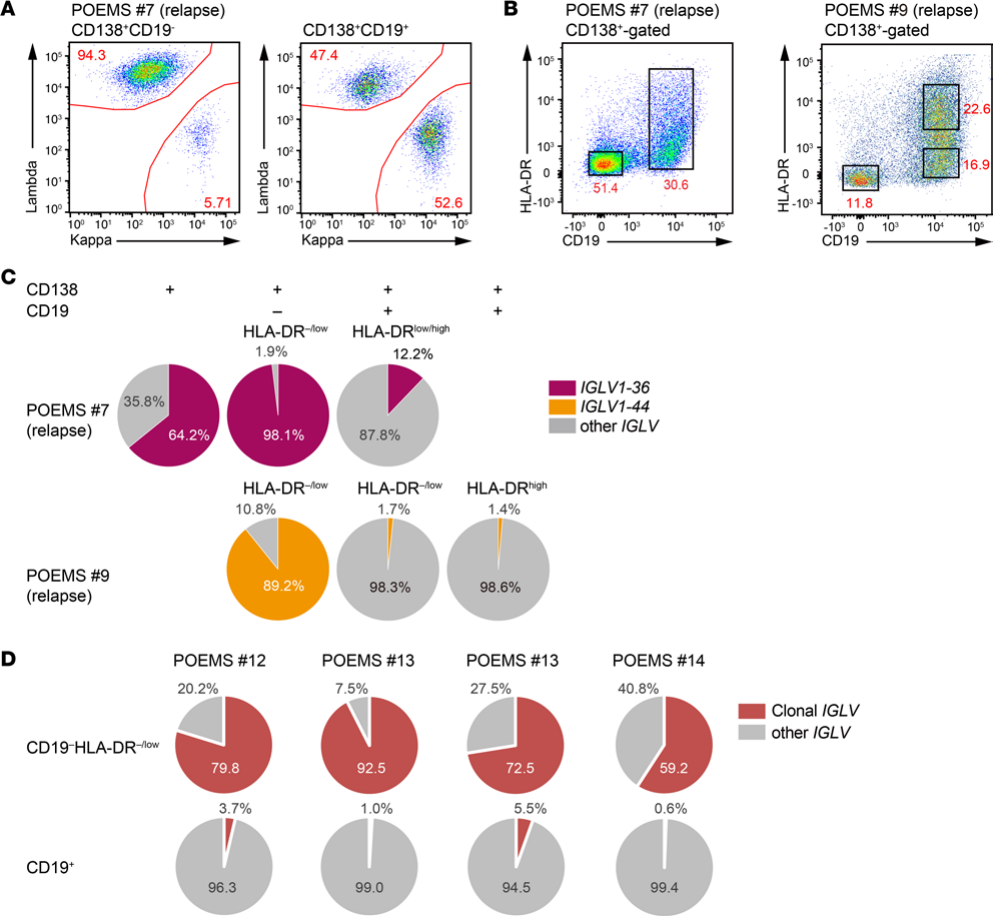
Immunophenotypic enrichment of POEMS clones. (**A** and **B**) Enrichment of POEMS clones expressing the clonal λ light chain in the CD138^+^CD19^–^ plasma cell fraction. (**A**) BM mononuclear cells (from patient no. 7 with POEMS at relapse) were stained for CD138, CD19, HLA-DR, and cytoplasmic light chains. (**B**) Cells from patients no. 7 and no. 9 with POEMS were stained for CD138, CD19, and HLA-DR. Boxes indicate sorting gates. (**C**) Frequencies of the POEMS-specific *IGLV1-36* and *IGLV1-44* sequences in all *IGLV* reads in the RNA-Seq data of the indicated plasma cell fractions. (**D**) Frequencies of the POEMS-specific sequences in all *IGLV* reads in the RNA-Seq data of the indicated plasma cell fractions in newly diagnosed POEMS patient samples.

Based on the results showing that POEMS clones were CD19^–^ and expressed low levels of MHC-II, we attempted to isolate POEMS clones from BM cells derived from patient no. 7 with POEMS at relapse for the third time (Supplemental Table 11). A flow cytometric analysis confirmed that the clonal cytoplasmic λ chain was enriched in the CD138^+^CD19^–^ fraction but not in the CD138^+^CD19^+^ fraction (Figure 6A). We then purified CD138^+^CD19^–^HLA-DR^–/lo^ and CD138^+^CD19^+^ plasma cells by cell sorting (Figure 6B). RNA-Seq analysis revealed that CD138^+^CD19^–^HLA-DR^–/lo^ plasma cells exclusively expressed *IGLV1-36* specific to patient no. 7 with POEMS, while CD138^+^CD19^+^ plasma cells showed only the mild enrichment of *IGLV1-36* (Figure 6C). Patient no. 9 with POEMS at second relapse (Supplemental Table 11) also showed enrichment of *IGLV1-44* in CD138^+^CD19^–^HLA-DR^–/lo^ plasma cells. We then validated these findings using 4 samples from patients with POEMS. As expected, we succeeded in prospectively purifying POEMS clones in the CD138^+^CD19^–^ fraction that expressed low levels of HLA-DR (Figure 6D and Supplemental Table 11).

**Figure 7:**
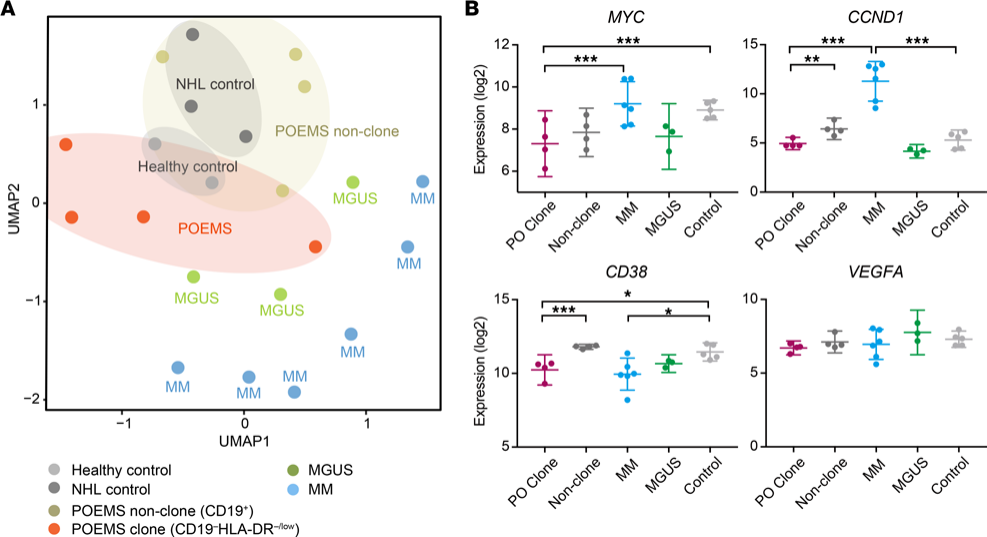
Transcriptional characteristics of enriched POEMS clones. (**A**) UMAP plots illustrating the transcriptomic profiles of control, POEMS clone and nonclone, MGUS, and MM plasma cells. Raw read counts of sorted CD138^+^ plasma cells (POEMS CD19^–^HLA-DR^–/lo^ [POEMS clone enriched], POEMS CD19^+^ [POEMS nonclone enriched], MGUS, MM, healthy BM, and non-Hodgkin’s lymphoma [NHL] BM) were normalized, batch effects were removed, and then counts were subjected to UMAP analysis. In order to increase the numbers of samples, we combined our previous data from 3 NHL controls, 3 patients with MGUS, and 3 MM patients with (16). (**B**) Expression of representative genes associated with the pathogenesis of POEMS syndrome or MM. The signal levels of the genes in normalized read counts (log_2_) are shown. Controls consist of 2 healthy BM controls and 3 NHL BM controls. Data are shown as the mean ± SEM. Adjusted *P* values: *P < 0.05, **P < 0.01, ***P < 0.001 by DESeq2.

We then performed detailed profiling of transcriptomes in POEMS clones using sorted plasma cells. UMAP plots clearly confirmed that CD138^+^CD19^–^HLA-DR^–/lo^ POEMS clones have unique expression profiles, which are different from those of MGUS and MM clones (Figure 7A). These data are consistent with those obtained by scRNA-Seq analysis. CD138^+^CD19^–^HLA-DR^–/lo^ POEMS clones again showed lower levels of *MYC* and *CCND1* than MM clones and expressed lower levels of *CD38* than control plasma cells, similar to MM clones (Figure 7B). *VEGF* mRNA was not upregulated in POEMS clones (Figure 7B). Among genes specifically upregulated in POEMS clones by scRNA-Seq, upregulation of *CFI* and *MUC1* was also confirmed in CD138^+^CD19^–^HLA-DR^–/lo^ POEMS clones but not others, such as *SPP1*, *SERPING1*, *C2*, and *HBB* (Supplemental Figure 7).

Finally, we evaluated whether our findings on POEMS clones are reproducible even when adopting different control plasma cells. We purified CD138^+^ plasma cells from BM of 2 healthy volunteers and subjected them to RNA-Seq. We then defined DEGs that were significantly differentially expressed among POEMS, MGUS, or MM plasma cells compared with healthy BM plasma cells (*q* < 0.0001). Using 1260 newly defined DEGs, we compared the transcriptomes of control plasma cells, POEMS clones (CD138^+^CD19^–^HLA-DR^–/lo^), POEMS nonclones (CD138^+^CD19^+^), MGUS, and MM plasma cells. As illustrated in UMAP plots (Supplemental Figure 8), POEMS clones were clearly separated from healthy BM plasma cells, MGUS, and MM. Of note, control plasma cells used for scRNA-Seq in this study, which were obtained from patients with early-stage lymphoma without BM invasion, showed similar expression profiles to those of BM plasma cells from healthy volunteers as well as control BM plasma cells from patients with non-Hodgkin’s lymphoma that we used in our previous study (16).

## Discussion

In the present study, we successfully identified POEMS clones in 5 of 10 patients with POEMS syndrome using scRNA-Seq, which discriminated POEMS clones from nonclones for the first time to our knowledge. The proportion of POEMS clones among total BM plasma cells was much smaller than that of MM and MGUS clones, as we had expected. This explains the difficulty we had faced in our previous studies using bulk BM plasma cells to get to the heart of the unique transcriptional features of POEMS clones (16). Our single-cell study confirmed that POEMS clones frequently have amino acid changes in the CDR1 and FR2 regions of *IGLV1-40* and *IGLV1-44* (15). In addition, we found a similar amino acid change in *IGLV1-47* (patient no. 8 with POEMS) and a novel change in *IGLV1-40* (patient no. 5 with POEMS). The amino acid changes at position 40 were always asparagine, whereas those at position 38 were variable. In spite of the varying amino acid changes at position 38, the amino acids changed to nonpolar amino acids in all cases, indicating that the protein structures around the regions were similar in POEMS syndrome and were associated with its pathogenesis.

Single-cell transcriptomes revealed unique transcriptional signatures of POEMS clones that were distinct from those of MM or MGUS clones. Protein synthesis–associated genes were upregulated in POEMS clones compared with control plasma cells, whereas UPR-associated genes were downregulated. These results suggest that POEMS clones are capable of hyperproduction of monoclonal Igs without the induction of high-grade UPR. This may explain why POEMS clones with smaller size than MM and MGUS can produce detectable levels of serum M proteins. In this regard, proteasome inhibitors, which are widely applied for MM therapy, could also target POEMS clones with excessive protein production, and thus, they should be tested for their therapeutic efficacy against POEMS syndrome.

Downregulation of MHC-II genes is another unique feature of POEMS clones. A recent analysis revealed that double-positive T cells with effector memory phenotype and PD-1^+^–exhausted CD4^+^ T cells were expanded and naive CD4^+^ T cells were decreased in the BM of individuals with POEMS syndrome (24). These findings suggest that CD4^+^ T cells are activated in the BM; therefore, downregulation of MHC-II on POEMS clones may contribute to the escape from T cell–mediated immune responses/surveillance. Reduced expression of MHC-II may also impair the recognition of POEMS clones by CD40L-presenting CD4^+^ T cells, resulting in attenuated CD40 signaling in POEMS clones. Expression levels of MM-related genes such as *MYC* and *CCND1* in POEMS clones were similar to or lower than those in control plasma cells. These results indicate attenuated proliferative capacity of POEMS clones and, thus, may explain the smaller size of POEMS clones compared with MM clones. *VEGF* was not elevated in both clonal and nonclonal plasma cells in POEMS syndrome, which provides the first direct evidence to our knowledge that VEGF is not produced by plasma cells in POEMS syndrome, and, therefore, the source of VEGF remains unknown.

We also found increased levels of OPN in the serum of all patients with POEMS syndrome analyzed. Expression of *SPP1*, encoding OPN, was upregulated in POEMS clones from 2 of 5 patients in scRNA-Seq analysis (Supplemental Figure 6). *SPP1* expression in sorted CD19^–^HLADR^–/lo^ POEMS clones was not significantly upregulated (Supplemental Figure 7). These data raise the possibility that the major source of OPN in POEMS syndrome is not POEMS clones, and upregulation of *SPP1* in POEMS clones may be the consequence of extrinsic factors. OPN regulates multiple steps of bone remodeling (25), and its serum level has been characterized as a biomarker of disease progression and bone destruction in MM (26). MM cells usually cause enhanced osteoclastic bone resorption; osteoclasts, in turn, produce OPN to enhance MM cell growth and survival and modulate osteoclast function (25, 27). In contrast, POEMS syndrome is characterized by osteosclerosis; therefore, the role of OPN in POEMS syndrome remains an intriguing issue to be clarified.

It is still unknown which factors contribute to the initiation of POEMS syndrome. The present study revealed an interesting correlation between POEMS clones and viral infections (Supplemental Table 9). Because chronic exposure to pathogens is associated with the progression of monoclonal gammopathy (28), it is possible that a specific viral infection might trigger the onset of POEMS syndrome. This hypothesis may also explain the lack of diversity in the structure of IgL in POEMS syndrome. Further studies are needed to address this hypothesis.

Based on the single-cell transcriptome data, we discovered that CD138^+^CD19^–^HLA-DR^–/lo^ is a general immunophenotype of POEMS clones. This result was consistent with previous findings showing that plasma cells with low CD45 expression levels had an abnormal loss of CD19 expression and a monotypic staining pattern for the cytoplasmic λ chain, while plasma cells with relatively stronger CD45 expression expressed CD19 and showed a polytypic staining pattern for cytoplasmic light chains in POEMS syndrome (8). In this study, we further clarified that POEMS clones downregulate the expression MHC-II genes. Thus, POEMS clones can be enriched in the CD45^lo^CD138^+^CD19^–^HLA-DR^–/lo^ fraction. Identification of the POEMS clone–specific surface marker phenotype allows us to directly access POEMS clones for further characterization of this poorly understood plasma cell disorder and monitor the therapeutic responses.

The small number of clones detected is the major limitation of this study. Recently, new technologies have been developed that can profile transcriptome and B cell receptor repertoire at the single-cell level. Our findings in this study could be extended by using these new technologies. However, we succeeded in prospectively enriching POEMS clones in the CD138^+^CD19^–^HLADR^–/lo^ fraction. The analyses using these sorted cells compensated for the limitation of scRNA-Seq approach in this study. Collectively, the combination of new scRNA-Seq technologies and our approach to enrich POEMS clones may further promote the characterization of POEMS clones and enhance our understanding of the pathogenesis of POEMS syndrome to develop novel therapeutic approaches.

## Methods

### Patients.

Patients with newly diagnosed or relapsed POEMS syndrome (*n* = 11) and newly diagnosed patients with MGUS (*n* = 2), MM (n = 2), or early-stage lymphoma without BM invasion, who were used as controls (*n* = 2) at Chiba University Hospital were enrolled in the present study (October 2016 to October 2020).

### Isolation of BM plasma cells.

BM aspirates were collected in heparin-containing tubes, and mononuclear cells were isolated with the Ficoll Paque Plus kit (GE Healthcare). Mononuclear cells were enriched for CD138 with magnetic beads (Miltenyi Biotec) and then stained by CD3 (HIT3a), CD11b (ICRF44), and CD138 (MI15) (BioLegend). CD3^–^CD11b^–^CD138^+^ plasma cells were sorted on a FACSAria III (BD).

### Flow cytometric analysis and cell sorting.

BM plasma cells enriched for CD138 with magnetic beads were stained with CD138, CD19 (HIB19), and HLA-DR (L243) (BioLegend) and analyzed on a FACSAria III. The detection of cytoplasmic κ and λ light chains in plasma cells was performed at SRL Inc., as previously described (29).

### scRNA-Seq analysis.

Sorted plasma cells were harvested and used in scRNA-Seq analyses with the C1 system (Fluidigm), because it is able to cover full-length transcriptomes and it provides the full sequences of Ig heavy and light chains (*IGH* and *IGL/K*). RNA-Seq libraries were constructed according to the manufacturer’s instructions as follows. Briefly, 96 cells were captured in flow cells and separated into independent chambers. First-strand cDNA was synthesized and further amplified using the SMART-Seq v4 Ultra Low Input RNA Kit for Sequencing (Clontech). Illumina sequencing libraries were constructed using the Nextera XT DNA Sample Preparation kit (Illumina). After an evaluation of the quality and quantity of the constructed RNA-Seq libraries using a BioAnalyzer (Agilent Technologies), sequencing was performed on the HiSeq3000 platform with a 100–base paired–end read. TopHat (version 2.0.13; with default parameters) was used for mapping to the reference genome (UCSC/hg19) and stringTie (version 1.3.4) for assembling and quantitating full-length transcripts with annotation data from iGenomes (Illumina). Read count matrices for genes and transcripts were generated using prepDE.py, and DESeq2 in R was used to normalize the expression count and analyze DEG.

### RNA-Seq.

Total RNA was extracted using the RNeasy Plus Micro Kit (Qiagen) and subjected to reverse transcription and amplification with the SMARTer Ultra Low Input RNA Kit for Sequencing (Clontech). After sonication with an ultrasonicator (Covaris), the libraries for RNA-Seq were generated from fragmented DNA with 8 cycles of amplification using the NEB Next Ultra DNA Library Prep Kit (New England BioLabs). After the libraries had been quantified using a bioanalyzer (Agilent), samples were subjected to sequencing with Hiseq2500 (Illumina) with a 60-base single read. TopHat2 (version 2.0.13; with default parameters) and Bowtie2 (version 2.1.0) were used for alignment to the reference human genome (UCSC/hg19). Normalization of the count value and significant expression differences were detected using DESeq2, with raw counts generated from StringTie.

### Analysis of the scRNA-Seq data.

The average expression level of a given gene was calculated as an average of the population of cells. Relative divergence was calculated as a SD divided by the average gene expression level. The significance of differences was assessed by the indicated methods. Regarding clustering, a hierarchical clustering program in the bioconductor package of R was used. *t*-SNE was performed using the R package Rtsne to reduce dimensions. We performed a GSEA as described previously (30). We excluded genes with no reads in both samples from GSEA comparison. Functional profiling of DEGs (*P* < 0.01) in POEMS clones and nonclones (Supplemental Tables 7 and 8) was performed on the g:Profiler website (31). Translocations, including t(11;14), t(4;14), and t(14;16), were examined by Genomon-SV (https://github.com/Genomon-Project/GenomonSV/tree/devel/genomon_sv).

### IG repertoire analysis.

V(-D)-J and CDR3 sequences in *IGH* and *IGL* genes were extracted from raw fastq files using MiXCR (version 2.0).

### ELISA for OPN.

Serum levels of OPN were measured using the Human Osteopontin Duoset ELISA kit (R&D Systems) according to the manufacturer’s instructions. In all patients, OPN was measured in sera cryopreserved at diagnosis. A standard curve was created by reducing data using computer software (GraphPad Prism) capable of generating a 4-parameter logistic curve fit.

### Data availability.

scRNA-Seq and RNA-Seq data obtained in this study were deposited in the DNA Data Bank of Japan (accession JGAS000289).

### Statistics.

Two groups were compared using the unpaired 2-tailed Student’s *t* test. More than 2 groups were compared using 1-way ANOVA. Data are shown as the mean ± SEM. *P* values of less than 0.05 were considered significant. Significant expression differences in RNA-Seq data were detected using DESeq2, and significance was presented as adjusted *P* values.

### Study approval.

The present study was approved by the ethics committee of the Chiba University Graduate School of Medicine (approval 1058) and The Institute of Medical Science, The University of Tokyo (approval 2020-30-0917). All patients agreed to participate in the study after informed consent following the guidelines of the Declaration of Helsinki.

## Author contributions

YI, MO, NM, and AI conceived the project and designed the study. YI, MO, NM, KK, YMN, and AI analyzed the data and prepared the manuscript. YNT, MS, and YS performed RNA-Seq. TM, ST, YT, CO, SM, SK, ES, and CN managed care for patients, collected BM samples, and analyzed clinical data. JI performed the pathological analysis. TK, EI, and MS assisted with the bioinformatics analysis. YI, MO, and NM contributed equally to this work.

## Supplementary Material

Supplemental data

Supplemental tables 1-11

## Version 1 09/21/2022

In-Press Preview

## Version 2 10/24/2022

Electronic publication
