# Supplementary material for: Unraveling unique features of plasma cell clones in POEMS syndrome with single-cell analysis
Source: JCI Insight. 2022 Oct 24;7(20):e151482. doi: 10.1172/jci.insight.151482 (PMC9714781; doi:10.1172/jci.insight.151482)
Supplement: Supplemental data [file jciinsight-7-151482-s133.pdf]

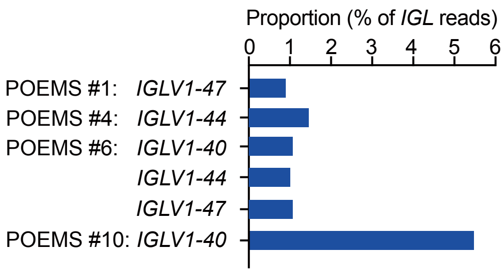


**Supplemental Figure 1. Frequencies of POEMS-specific *IGL* sequences in POEMS 1, 4, 6, and 10**
Frequencies of POEMS-specific *IGL* sequences in the RNA-seq of 200 plasma cells from POEMS 1, 4, 6, and 10 who did not have any candidate POEMS clones in the scRNA-seq analyses.

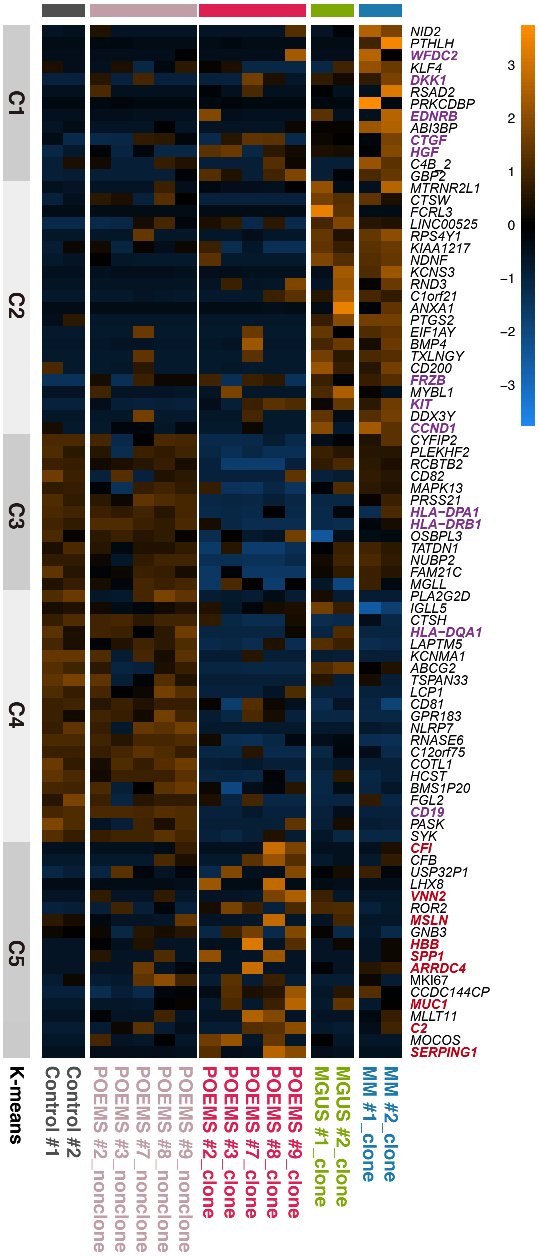


**Supplemental Figure 2. Gene expression profiles of POEMS clones and non-clones.**
K-means clustering of scRNA-seq data. Normalized read counts (log2) were averaged within each group and transformed into z-scores. Heatmap of normalized expression levels were shown with same gene sets and order as shown in Figure 3A.

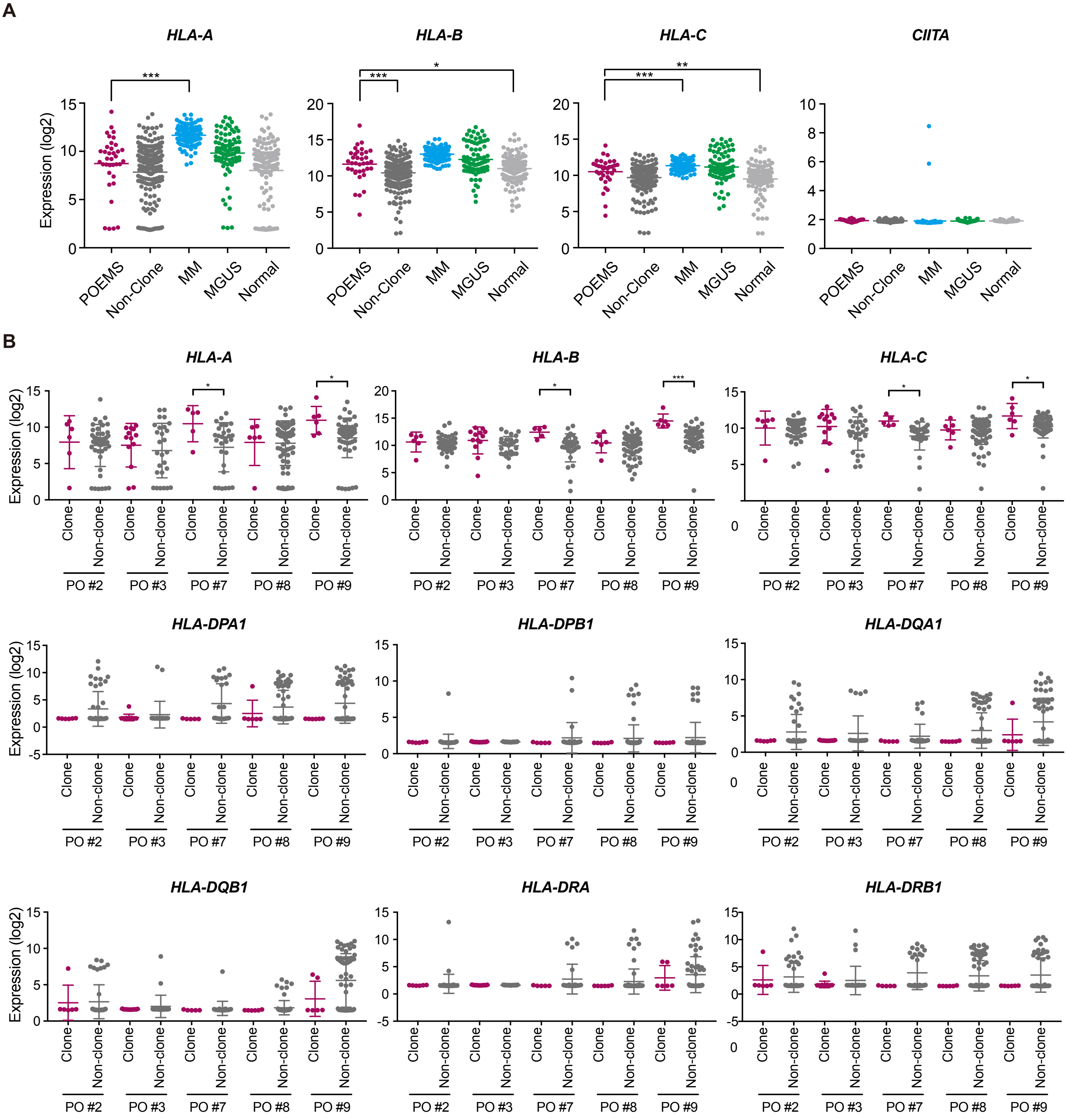


**Supplemental Figure 3. Gene expression profiles of POEMS clones and non-clones.**
Expression of MHC class I and *CIITA* genes in normalized read counts of scRNA-seq data (log2) in POEMS clones and non-clones in total (A) and in each POEMS patient. Adjusted p values: *, $p < 0.05$; **, $p < 0.01$; ***, $p < 0.001$.

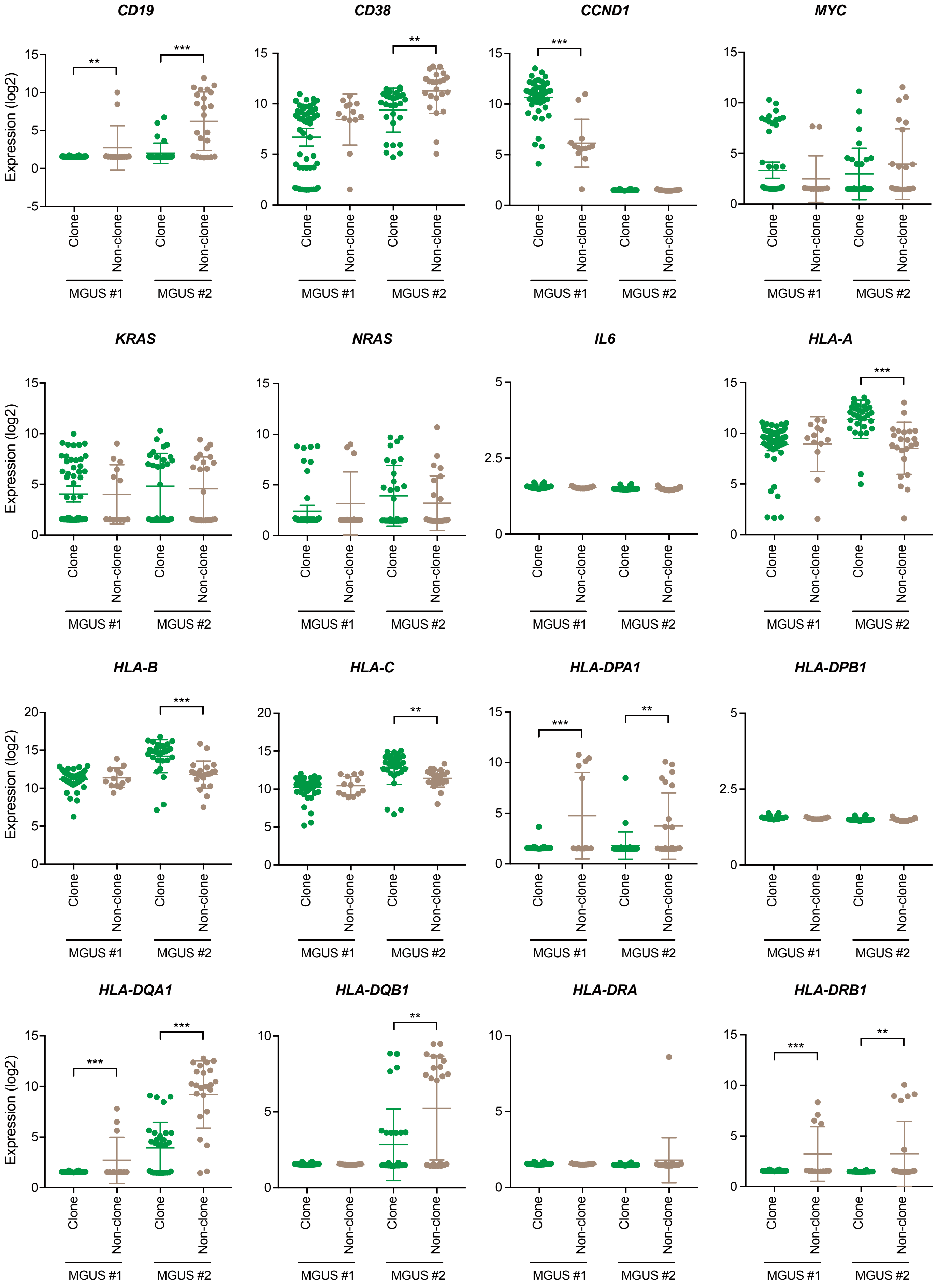


**Supplemental Figure 4. Expression of genes in MGUS clones.**
Expession of genes in plasma cells from MGUS patient #1 and #2. Normalized read counts of scRNA-seq data (log2) are shown. Mean values are indicated by horizontal bars. *, $p < 0.05$; **, $p < 0.01$; ***, $p < 0.001$ by the Student's *t*-test.

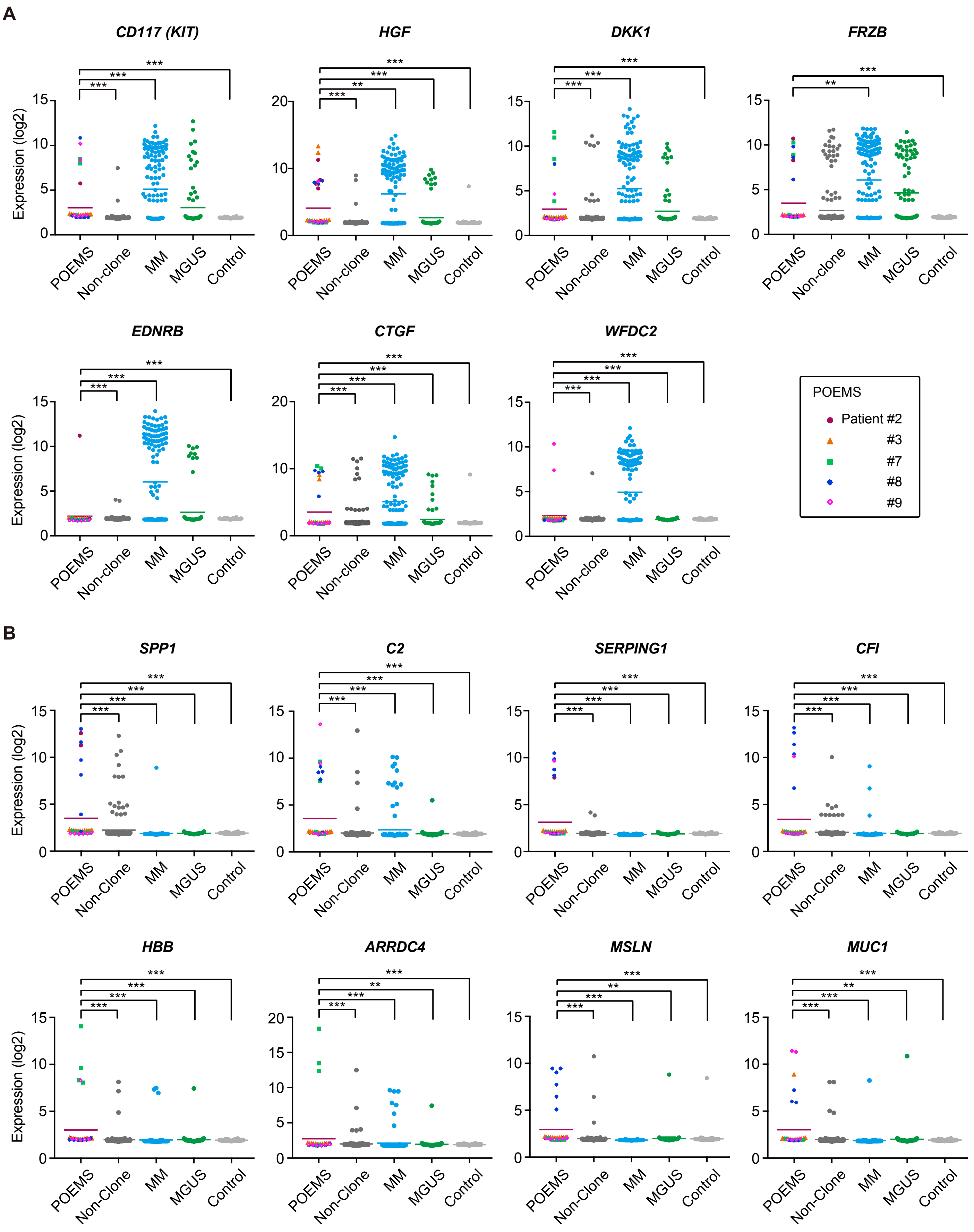


**Supplementary Figure 5. Expression of genes up-regulated in MM and POEMS clones.**
Expression of genes prefentially up-regulated in MM (**A**) and POEMS syndrome (**B**). Normalized read counts of scRNA-seq data (log2) are shown. Mean values are indicated by horizontal bars. Adjusted p value: *, $p < 0.05$; **, $p < 0.01$; ***, $p < 0.001$.

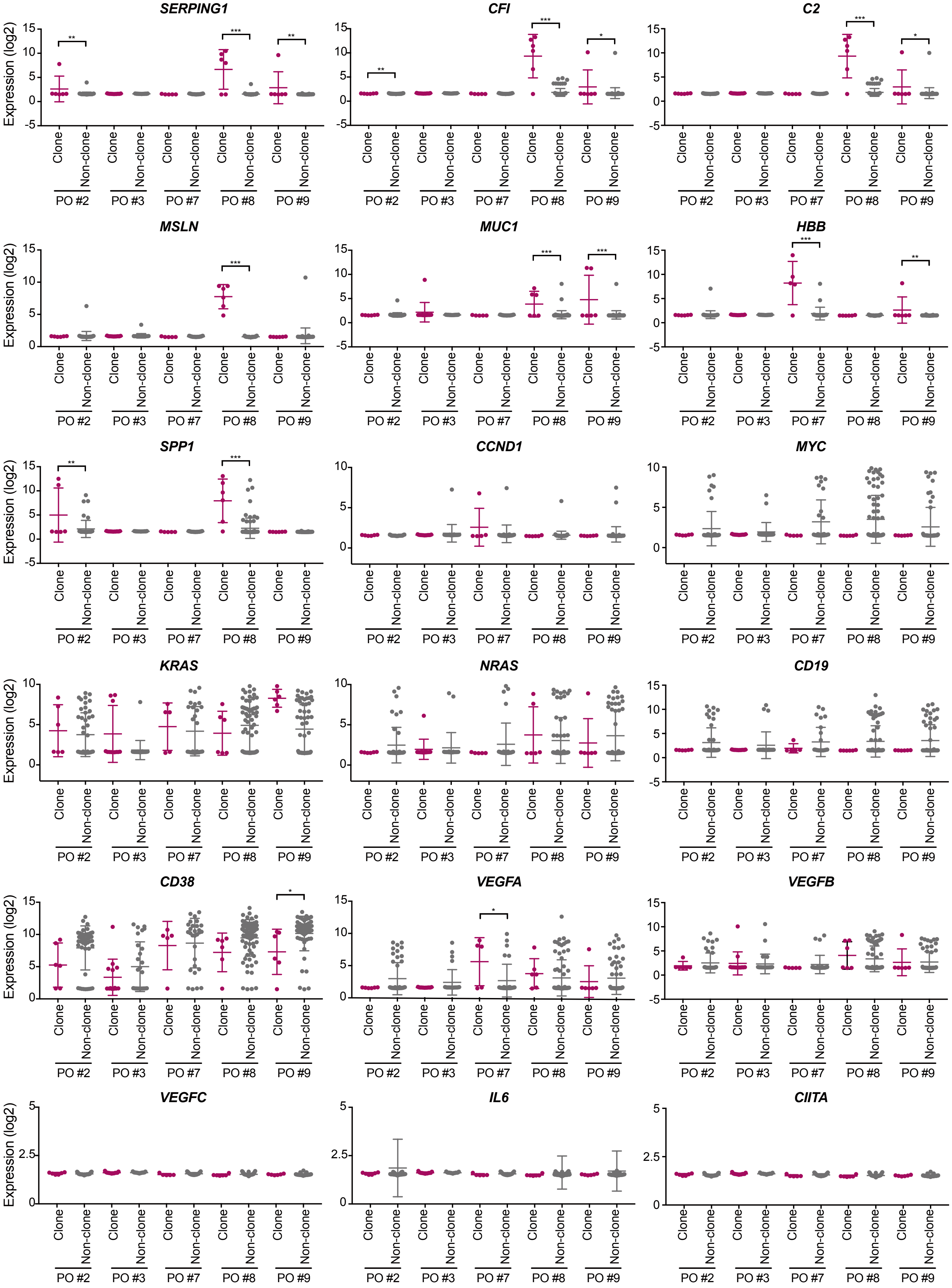


**Supplemental Figure 6. Gene expression profiles of POEMS clones and non-clones**

Expession of genes in plasma cells from each POEMS clone and non-clone. Normalized read counts of scRNA-seq data (log2) are shown. Mean values are indicated by horizontal bars. *, $p < 0.05$; **, $p < 0.01$; ***, $p < 0.001$ by the Student's *t*-test.

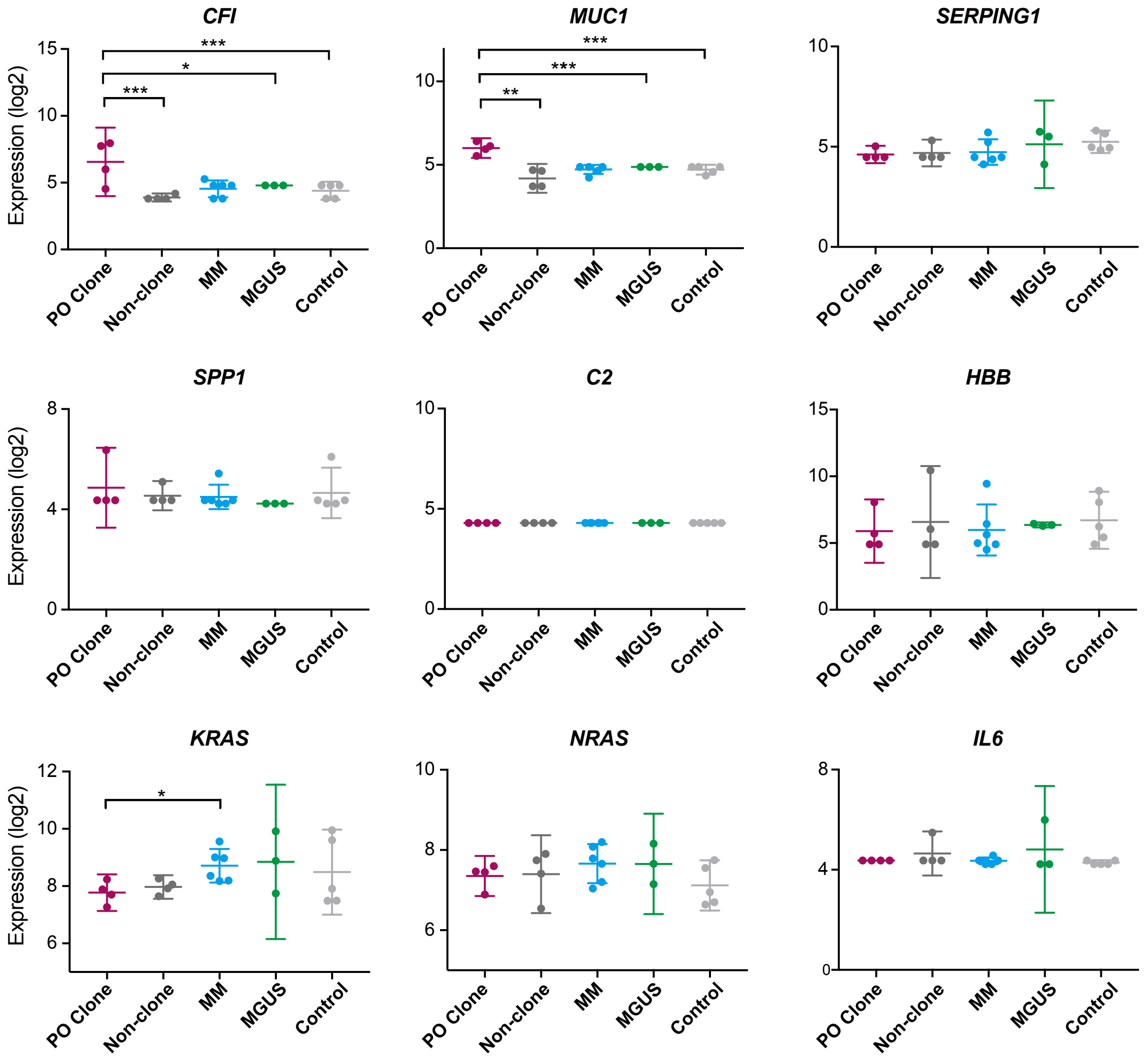


**Supplemental Figure 7. Gene expression profiles of enriched CD19⁻HLA-DR$^{lo}$ POEMS clones by cell sorting**
Expession of genes in CD138$^{+}$CD19$^{-}$HLA-DR$^{low}$ (POEMS clones-enriched) and CD138$^{+}$CD19$^{+}$ (POEMS non-clone enriched) plasma cells and CD138$^{+}$ MGUS and MM plasma cells. Normalized read counts of RNA-seq data (log2) are shown. Data are shown as the mean ± SEM. MGUS data was retrieved from Nagao et al., Leukemia 33:1723-1735, 2019. Adjusted p value: *, $p < 0.05$; **, $p < 0.01$; ***, $p < 0.001$.

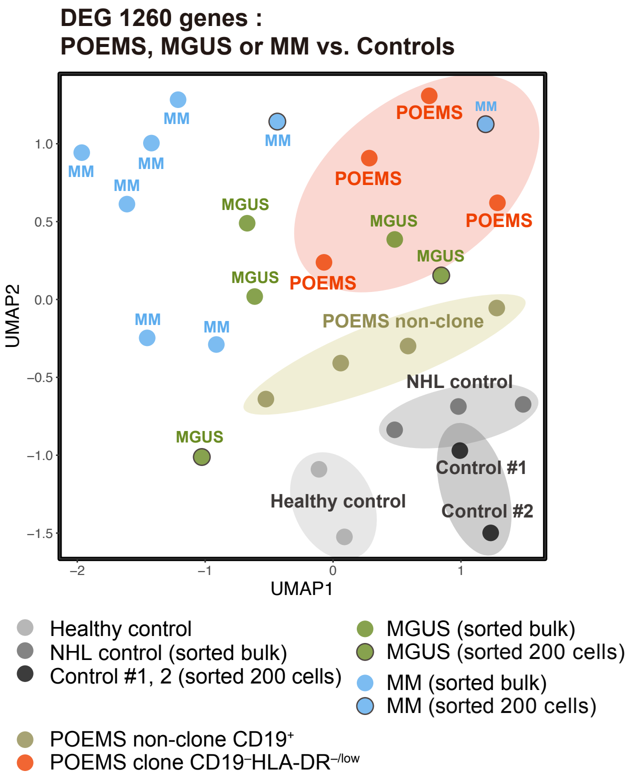


**Supplemental Figure 8. Gene expression profiles of control plasma cells**
UMAP plots illustrating the transcriptomic profiles of Control, POMES clone and non-clone, MGUS, and MM plasma cells. Bulk RNA-seq data of $CD138^{+}CD19^{-}HLA\text{-}DR^{low}$ (POEMS clone-enriched) and $CD138^{+}CD19^{+}$ (POEMS non-clone-enriched) plasma cells, $CD138^{+}$ MGUS and MM plasma cells, 200 plasma cells from Controls (#1 and #2), MGUS (#1 and #2), and MM (#1 and #2) in Figure 1B were normalized and removed batch effects, then differential expression analysis of read counts were performed. A total of 1,260 DEGs between POEMS, MGUS, or MM compared with healthy BM plasma cells were obtained using the cutoff value q < 0.0001. UMAP analysis was performed using DEGs. Data of 3 NHL BM control plasma cells, 3 MGUS plasma cells, and 3 MM plasma cells were retrieved from Ref. 16 (Nagao et al., Leukemia 33:1723-1735, 2019).
